# Supplementary material for: An affordable solution for investigating zebra finch intracranial electroencephalography (iEEG) signals
Source: PLoS One. 2025 Oct 3;20(10):e0322951. doi: 10.1371/journal.pone.0322951 (PMC12494284; doi:10.1371/journal.pone.0322951)
Supplement: Attachment — Submitted filename: review_of_PLOS_PONE_IEEG_finch.pdf [file pone.0322951.s002.pdf]

The paper by Abolghasemi and colleagues describes the use of open source brain computer interface technology (OpenBCI)to carry out intracranial EEG recordings in the songbird. The authors note that a demonstration of a cheap technology that enables recording structured brain activity, as promised by this approach, would increase access to experimentation for laboratories that do not have the resources or expertise to use more state of the art techniques such as electrophysiological recordings or calcium imaging.

Beyond this reasonable point, it also seems possible that a simple and cheap description of how EEG recordings can be made in the songbird to evaluate distributed brain activity in the context of different conditions could be useful for many laboratories to augment other techniques.

I think that with appropriate modifications and additions to address concerns and suggestions as outlined below, the manuscript could be appropriate for publication in PLOS One.

In brief, these suggestions are three-fold 1) In order to achieve the authors' intent of making this an easily access accessible technology, there are numerous places where more detail is needed in the methods and/or the accompanying point-by-point online protocol to make the approach clear and reproducible.

2) the protocols.io description in S1 file contains a lot of specific information about the bird procedures such as how to induce anesthesia, sterilize instruments, restrict food, monitor recovery, house birds, prepare incision site, hold bird during experiment, etc. Much of this level of detail regarding surgical procedures, animal handling, animal housing, etc, is subject to review by each institutions animal care and use committee. It needs to be made clear that much of this detail is subject to approval of the relevant IACUC (see further comments below)

3) beyond technical aspects of the protocol, it would be useful if the authors could make some additional measurements to assess the nature of the signals that can be recorded and to compare those with the kind of data that have been recorded in previous work in songbirds. I particularly recommend looking at the work from Low and Margoliash who used iEEG recordings to characterize sleep states in zebra finches. It would be a relatively straightforward and informative experiment to record during sleep in zebra finches and determine whether or not the signals agree with those reported by Low and colleagues. Beyond that, anything further that could be done to help understand the nature of the recorded activity, and factors, such as position of electrodes, that affect those signals would be helpful in assessing for what applications this kind of system might be used. Because this article is intended as a protocol rather than presenting a research finding or data set, I think that such additions are optional and at the discretion of the authors, but I think they would significantly strengthen the manuscript.

More details/suggestions on these points:

1) For a paper that is primarily intended to provide a protocol for the use of OpenBCI resources for iEEG recordings, you should provide significantly more detail within the paper and/or at protocols.io on how to get the system working, the specific modifications for use in the zebra finch, and any issues that have arisen with respect to troubleshooting or analysis. As much as possible, you should make this a resource for someone to assess whether this is the right technology for them to use, and to glean most of the information they need to implement what you have done.

This includes:

a) perhaps in introduction and/or in the methods (by Methods here and in the rest of the review I am referring both to the methods section and to the protocols.io description), please provide a general description of Open BCI and some relevant citations or references to where one can go online to procure

the relevant hardware and software to reproduce the authors' system. The paper itself lists reference 24 as having some of this information, but no reference 24 is included in the bibliography. More generally, at several points the authors say they followed procedures as described in some other reference. It is fine to direct people to a reference for further details, but please at least describe enough within the methods of this paper itself so that readers have an understanding of the relevant details. If Open BCI has different versions of hardware and/or software, please make note of this and detail which were used in this study; if there are choices here for different applications, perhaps include a table or further description that both specifies what was used here and what other options might be available.

These additions are to fully orient readers to the general resources that were already available that you have used in the study and that readers could access.

b) Some aspects of the study will be specific to your modifications for use in songbirds, and this should also be more clearly spelled out so that others do not have to reinvent what you have done. For example, please specify in more detail how the electrodes were constructed (perhaps with photo) and perhaps anything further that you can note about whether or not the properties of construction matter. Were these electrodes that were provided by Open BCI or another commercial source, or did you construct them? What was the material, what was the tip exposure like, what was the impedance? Protocols.io is a good place for much of this, so please make sure to direct the reader clearly to that resource for information.

It would be very helpful to specify some further detail, perhaps showing more specifically a parts list of materials acquired from OpenBCI, and additional materials that you needed to add to run these experiments, and this could be worth putting in a table or figure.

c) How many electrodes were placed in or on the brain, where were they placed, and how much does it matter? I could find no detail either on the general placement electrodes, or how they contacted the brain. For example, were these resting on the meninges? Or were the meninges removed, or were these sitting on the inner layer of skull, etc? Was any kind of conductive material used to enhance conductivity and signal-to-noise ratio? How are they affixed to the brain? At one point it was mentioned that there is a cerebellar reference electrode. Where and how was that placed, and how were the signals from each of the other electrodes connected to the amplifiers/hardware provided by Open BCI?

d) with respect to electrode placement, please clarify how the tips of the electrodes were positioned with respect to relevant landmarks such as the bifurcation of the mid-sagittal sinus. Were all electrodes referenced to the cerebellum, or were there pairs of electrodes across which differential signals were compared?

e) with respect to electrode placement, can the authors provide any guidance on how much it matters? For EEG, usually there is quite a bit of dependency of signals on electrode placement. The authors show data from "anterior" and "posterior" electrodes, but do not specify the coordinates where these were placed. We can see that there are some differences between the anterior and the poster electrodes in terms of the signals that are recorded. However, it would be helpful if the authors could provide some more sense of the spatial variation in signals that would help readers understand what kind of spatial resolution is there for distinguishing signals between different brain areas: The authors describe one of the advantages of iEEG as potentially providing a greater "survey" of activity distributon across the brain than is normally achieved with electrophysiology. This would be substantiated and clarified to the extent that signals from nearby recording locations look different from each other.

f) It would be useful to sketch out more clearly any additional workflow that the authors can provide, for example with respect to how the signals were recorded, and what software was used to process them, etc., so that readers could take advantage of the protocol without needing to reinvent various components or go

to other sources. If these components are all provided within Open BCI, that should be clarified with appropriate references.

2) For the specifics of experimental results that are shown in figures, please also consider where more detail might be helpful for a reader. This again includes some aspects of methods that should be in this paper and not included by reference. For example, for suppression duration shown in figure 2, how was this measured? For low medium, and high levels of isofluorane, how long were animals at a fixed level of anesthesia before measurements were made? For figure 3, is this the average of some number of stimulus presentations or a single trial? What was the stimulus duration in these figures? The stimulus duration is particularly important to show, because of the structure of responses that show, for example, some peak at 1.1 seconds - does that relate in any way to when the stimulus stopped or changed in amplitude? Showing oscillograms of the stimuli here would be helpful and could also address one other question, which is how loud were the stimuli?

3) as noted at the top, the more that you can show that this set up records useful signals that are similar to those from other eeg recordings in birds, the more likely readers will follow your approach. In that respect, please look a little bit more for songbird references that have recorded EEG's. Low and Margoliash published a paper ("Mammalian-like features of sleep structure in zebra finches " PNAS 2008) that I tracked down, but I believe there may be others, including from Low. It would be worth citing this paper and comparing your approach with theirs; if you are in a position to do so, recording activity during sleep to see if you can generate signals like those shown in figure 2 (of Low), and spectra like those shown in figure 3 (differences between slow wave sleep and REM sleep.) would be a nice way of validating your EEG signals relative to those in the literature.

It is perhaps beyond the scope of what you might easily do, and is not necessary, but it would also be interesting to see if under light anesthesia you could get different responses to playback of the bird's own song (BOS) versus reversed (BOS); within the song system nuclei, including the superficial structure HVC, there is a greater response to BOS, and if you are able to detect tuning for song stimuli in this manner or others, that would also promote the utility of these measures for functional use.

Technical: in the versions that I downloaded for review, and the linked figure files, the resolution was very coarse and pixilated. I could not read the axis labels in figure 2, and many other details of the figures were blurry. Please check to make sure that the resolutions of final figures is improved.
Note also that Figure 1 was difficult to follow. It looks like there were four colored wires, sticking out in a pitchfork shape from something, but I couldn't tell what that thing was, and this is not described in the legend. The electrodes are shown as somehow spread out outside of the bird's brain, but sticking up quite a bit.

I have outlined below some more specific suggestions for organizing the protocols.io description. Some of these are redundant with comments above – I was unable to access the protocols.io material from the supplementary materials link, and so wrote the initial review without access to that material. When I was able to access the prorocols.io description, there was something off with the formatting – some sentences were cut off, some strange symbols appeared. I thought the main text was generally well written, but found much of the wording in the prorocils.io to be confusing and to have many typos and grammatical errors, including lots of missing punctuation, and it would benefit from careful proofreading.

****

For the specific protocol, I suggest that you organize this in sections that separate several different components:

0. Overview of OpenBCI and what references readers should access if they are going to follow your protocol. What is provided by OpenBCI and what will readers additionally need to do to implement what you have done? A quick look at the open BCI website indicates that it provides a great deal of useful information. As part of the Over view of the protocol, it would be useful to sketch out what aspects of your recording apparatus and procedures directly use hardware, software, and resources that are well-described by OpenBCI, and what parts you have customized for application to the songbird. Where there are details of the protocol that follow specific step by step instructions form OpenBCI, you do not need to reproduce them, but within your own protocol, you should make clear that the reader should use a specific set of instruction or resources form OpenBCI whenever that is the case. For example, my comments suggestions below may include some things that are redundant with OpenBCI (Such as the pinout configuration, or recording software, to GUI, etc), but there is no clear reference to this in the protocol.io. Overall, it would be fine, if appropriate, to include a statement along the lines "For iEEG recording in the bird, we start with a basic EEG recording configuration that is detailed by OpenBCI. A materials list and step by step directions for this are provided on the OpenBCI website (URL:....). (Then explain what specific OpenBCI documents I need to access and what the goal of this aspect of the protocol is: (will I have a working system that can record EEG signals?, play stimuli? Carry out abalyses? Etc). Then something like: "We suggest readers that are interested in following our protocol first familiarize themselves with these materials from Open BCI before proceeding with customization of the system for songbird experiments as elaborated below"

I suggest providing a more complete description of the protocol and ordering it with clearly defined sections along the following lines (doesn't have to be exactly this, and some of the following may be achieved by specific reference to Open BCI materials, as noted above).

It would probably be useful to include a full parts list as a table in this section. IF some of these parts come as. Kit form Open BCI, versus needing to be purchased separately, that could be noted here.

1. **Assembling the recording apparatus** (this section could explain what kind of inputs and outputs the open BCI system provides for, and give some more specific guidance on how to assemble the relevant recording apparatus: this might include a schematic showing the pins on the open BCI device, and what their functions are (i.e. where should a reference ground be connected? how many EEG electrodes can be connected? into which pins? does this device itself provide stimulus output or is that something separate? if this device provides for output on what line does that output appear and how would you connect output to speaker or other? If an Opto-isolator is needed, how is that built, or where can it be bought? Schematically, where should it be connected? (Is any of this described on Open BCI site?).

What kind of power is required, and where should the power supply be connected to pins on the Open BCI board? (again, if some of this is explained as part of the Open BCI materails, it is fine to refer to that directly, but may still be useful to show us schematic diagram and/or photographs of how you have specifically configured things for the songbird).

2. **Electrode assembly**; useful to describe as you have done, but perhaps also to show pictures for the recording electrodes, and to make clear to which pins they should be connected. You could also discuss in this section alternative configurations, for example if readers wanted to record more or fewer sites. Is each electron ultimately referenced to the cerebellum? In that case, is the number of recording electrodes anywhere between one and four, or are more EEG electrodes possible? Are the cerebellar electrodes constructed in the same way as the recording electrodes? And are the two pictured cerebellar electrodes the same, just one on each side of the brain, or do they serve some different function? Are those two electrodes linked together if they provide a common ground or do they attach to the open BCI board at separate pins?

3. **Placement of electrodes** – this section could both note the specific locations where you placed electrodes for the experiments illustrated in the paper. But it could also discuss issues of placement, such as whether there are particular advantages to placing the electrodes at symmetric locations across the mid-sagittal plane, or any other information you may have gained in experimenting with different electrode locations. What works well and what doesn't? The Low and Margoliash paper that is mentioned in the main body of the review may also have some useful information on this.

4. **Recording iEEG signals and troubleshooting**; Does open BCI come with its own data acquisition software or is this something that is available on the GitHub repository that you list? the GitHub repository is currently empty via the link that I was provided, so I could not evaluate what is there. If there is an open BCI GUI or software for data acquisition, it would be useful to explain how this is set up for your experiments, perhaps also showing a screenshot of the GUI, and providing information about any relevant parameters that need to be set, and where and how files are recorded and can subsequently be opened for analysis. See also the last section about animal handling and procedures – as noted there, it will be important to provide further caveats about the necessity of proceeding only in ways that have been approved by each institutions animal use committee.

5. **Stimulus presentation and data synchronization**. Is stimulus presentation part of open BCI, or is this something that you did completely separately with an Arduino, etc.? Here it would also be useful to explain both how you presented stimuli, and how you synchronized presentation with data acquisition. For example, did your Arduino output a TTL pulse or other mark that was recorded on one of the channels of the Open BCI board? Or does the open BCI system drive the Arduino or otherwise control stimulus

presentation?

You could also include in this section something about how stimuli were generated – what kind of file formats – and how software is set up for this presentation. I don't think you need to go into this too much, as the requirements for different people using this apparatus will differ, but it is still useful to sketch out some of these details as they will provide a framework for people to modify in order to carry their own experiments.

6. **Data analysis**. At minimum a description of the file formats and how they can be opened would be useful. Does open BCI itself provide tools for doing things like averaging across presentations and carrying out spectral analysis, or is this something that needs to be done separately? In that case, does your repository include any tools for this, or is this something that people would need to program for themselves in Python or Matlab, etc.? For potential users, providing these details will be very useful and enabling.

7. **Troubleshooting**. no matter how good your directions, It is likely that things will go wrong for people in one way or another. If you have any advice based on your own experience about troubleshooting problems that have arisen, it could be useful to include some of that here. For example, you experienced some sort of noise issues that necessitated using an opto-isolator, and also a notch filter. Might these be things that are always needed, or perhaps only in cases where there is an unconditioned power supply, etc. Any tips you can provide here will be appreciated when people run into problems. In any of the above sections, it would be fine to direct people to relevant resources provided by open BCI. If there is an open BC manual that covers specific things, you could refer to that more explicitly so that people will know where to look, but I think it would also be good to summarize the points as enumerated above in your protocol itself.

8. **Bird handling and surgical procedures, etc**. This is an especially tricky one, but I think should largely be separated from the other sections and treated with care. The current protocol provides lots of details about things like food deprivation, bird handling, inducing anesthesia, placing the bird in a restraining tube, removing feathers, opening craniotomy, delivering analgesics and antibiotics post experiment, housing of birds after experiments, etc. At most institutions, all of these details will need to be approved by the institutional animal care and use committee, and the requirements will be different at different institutions. **Many of the specifics outlined here would not be allowed at my institution.** For example, I cannot use alcohol to sterilize electrodes, nor can I pluck feathers, but must instead cut them close to the scalp. We would need special permission in order to restrain birds in a tube as you have illustrated, and it might not be allowed. We normally have to provide heat during experiments and recovery. We might not be allowed to use the same antibiotics and analgesics that you list here, etc.

For all of these reasons, I think it would probably be easiest for you to write something more general about the animal handling and surgical procedures. You should certainly

note that any specifics need to be approved by each users institutional animal use committee. If you do that, I suppose you could outline the specifics of what you are allowed to do at your institution, but please make it clear that this might not be universally accepted. I am not sure for example that we would be allowed to use ear bars of the sort that you describe -- made from hypodermic needles, and the mask that we use for isoflurane anesthesia would need to be specifically approved by our veterinary staff , etc. The one thing that I think is worth making specific is exactly how your electrodes are placed - in this case, inserting them into the craniotomy, but then sliding them between dura and the skull. It would helpful to be similarly specific about how the cerebellar electrodes are placed.
