# Supplementary material for: An affordable solution for investigating zebra finch intracranial electroencephalography (iEEG) signals
Source: PLoS One. 2025 Oct 3;20(10):e0322951. doi: 10.1371/journal.pone.0322951 (PMC12494284; doi:10.1371/journal.pone.0322951)
Supplement: Attachment — Submitted filename: Response_to_the_reviewers_auresp_2.docx [file pone.0322951.s005.docx]

**Response to the Editor and Reviewers**

Dear Editor and Reviewers,

We would like to express our sincere gratitude for your thorough review and insightful comments. Your feedback has significantly enhanced the quality and clarity of our work. We carefully considered each suggestion and integrated them into the revised version of the manuscript. As noted by the editor, we compared our method with a gold standard in the zebra finch literature. To do so, we conducted a new experimental setting, presenting five different auditory stimuli—including BOS and reverse BOS—to zebra finches under light anesthesia while recording from the CMM and HVC brain areas. Our results align with previous findings in this area, which are elaborated in the manuscript.

We genuinely believe these improvements have strengthened our research, and we hope you will feel the same upon reviewing the updated manuscript.

In the following sections, we provide detailed responses to your comments, outlining the revisions and how they address the issues you raised.

Thank you once again for your time and consideration.

Sincerely,

*Milad Yekani, DVM, PhD*

*Postdoctoral Researcher*

*Institute for Research in Fundamental Sciences (IPM)*

*miladyekani@ipm.ir*

*+98 914 347 8916*

# Responses to reviewer 1’s comments

*1. For a paper that is primarily intended to provide a protocol for the use of OpenBCI resources for iEEG recordings, you should provide significantly more detail within the paper and/or at protocols.io on how to get the system working, the specific modifications for use in the zebra finch, and any issues that have arisen with respect to troubleshooting or analysis. As much as possible, you should make this a resource for someone to assess whether this is the right technology for them to use, and to glean most of the information they need to implement what you have done. This includes:*

*a) perhaps in introduction and/or in the methods (by Methods here and in the rest of the review I am referring both to the methods section and to the protocols.io description), please provide a general description of Open BCI and some relevant citations or references to where one can go online to procure the relevant hardware and software to reproduce the authors’ system. The paper itself lists reference 24 as having some of this information, but no reference 24 is included in the bibliography. More generally, at several points the authors say they followed procedures as described in some other reference. It is fine to direct people to a reference for further details, but please at least describe enough within the methods of this paper itself so that readers have an understanding of the relevant details. If Open BCI has different versions of hardware and/or software, please make note of this and detail which were used in this study; if there are choices here for different applications, perhaps include a table or further description that both specifies what was used here and what other options might be available.*

*These additions are to fully orient readers to the general resources that were already available that you have used in the study and that readers could access.*

**Response:**

**General description of the OpenBCI**:

We have added a description of OpenBCI. The OpenBCI Cyton Biosensing Board was used for this study, and we have provided relevant links for obtaining the hardware and software directly from the OpenBCI website. We have also described alternative hardware configurations, such as the Ganglion board (Protocol.io, Section 1, "Getting Started with OpenBCI"). We have also included a general description of OpenBCI in the introduction and provided related references. This introductory part mainly focuses on OpenBCI’s capabilities and hardware features.

Please refer to the fifth paragraph of the article's introduction: *“OpenBCI offers a versatile open-source hardware platform for various biosensing applications, including EEG, EMG, and ECG signals. At its core, the OpenBCI Cyton board integrates several key components: a PIC32MX250F128B microcontroller, a ChipKIT UDB32-MX2-DIP bootloader, an ADS1299 analog-to-digital converter with eight channels (expandable to 16) and a maximum sampling rate of 16 kHz, and a 3-axis LIS3DH accelerometer. Users can configure EEG channels in either monopolar or bipolar modes, while the board also accommodates five external digital inputs and three analog inputs. Moreover, the device supports data streaming over Wi-Fi via Transmission Control Protocol (TCP)”.*

**Step by step instruction for OpenBCI**:
To make it easier for readers to reproduce our setup, we have included step-by-step instructions in the **protocol.io description** on assembling and configuring the OpenBCI system for recording iEEG data. (Section 2, Assembling the Recording Apparatus).

*b) Some aspects of the study will be specific to your modifications for use in songbirds, and this should also be more clearly spelled out so that others do not have to reinvent what you have done. For example, please specify in more detail how the electrodes were constructed (perhaps with photo) and perhaps anything further that you can note about whether or not the properties of construction matter. Were these electrodes that were provided by Open BCI or another commercial source, or did you construct them? What was the material, what was the tip exposure like, what was the impedance? Protocols.io is a good place for much of this, so please make sure to direct the reader clearly to that resource for information. It would be very helpful to specify some further detail, perhaps showing more specifically a parts list of materials acquired from OpenBCI, and additional materials that you needed to add to run these experiments, and this could be worth putting in a table or figure.*

**Response:**

**Electrode preparation**: The new protocol describes how to prepare the electrodes. It includes steps for bending, shaping, and disinfecting the electrode before usage. Regarding the concerns about disinfection by ethanol, we have reviewed all procedures with our veterinarian. We were advised to avoid contact and ensure all ethanol was evaporated and rinsed with saline. We also reminded readers to consult their veterinarian, as these methods may not agree with their institute's regulations and protocols. See Protocol.io, Section 4, "Electrode Preparation".

**Electrode placement**: The protocol explains how electrodes are placed under the skull and on the dura mater and how force is applied to ensure they are correctly fixed. It also describes how electrodes are pushed into position, including a diagram showing electrode placement in relation to the skull and brain. See Protocol.io, Section 28, "Electrode Placement and Fixation."

**Parts list and materials**: A detailed list of materials includes stainless steel wire, hypodermic needles, dental cement, and other supplies needed to construct and implant the electrodes. It also specifies where to purchase these materials and necessary tools. See Protocol.io, "Materials".

**Electrode montage and recording setup**: This section outlines how electrodes are connected to the OpenBCI board and details the positions (e.g., cerebellum for reference electrodes). See Protocol.io, Section 7

**Electrode impedance**: In protocol.io, we have provided a comprehensive explanation of how the electrode's impedance was measured and its range (15k-20k). See Protocol.io, Section 8.

*c) How many electrodes were placed in or on the brain, where were they placed, and how much does it matter? I could find no detail either on the general placement electrodes, or how they contacted the brain. For example, were these resting on the meninges? Or were the meninges removed, or were these sitting on the inner layer of skull, etc? Was any kind of conductive material used to enhance conductivity and signal-to-noise ratio? How are they affixed to the brain? At one point it was mentioned that there is a cerebellar reference electrode. Where and how was that placed, and how were the signals from each of the other electrodes connected to the amplifiers/hardware provided by Open BCI?*

**Response:**

We have carefully addressed this comment and have added further details about the placement and contact of the electrodes in the protocol.io file. Specifically:

**Number of electrodes**: We clarified that **four electrodes** were used in our setup: two for signal recordings and two for reference and noise cancellation. These electrodes were positioned in both **frontal and auditory regions**, and the reference electrodes were located on the **cerebellum**. These placements have been specified in greater detail, including how their locations were determined using bifurcation (Protocol.io, Section 6, "Electrode Montage").

**Electrode contact with the brain**: We explicitly stated that the electrodes were placed **under the skull and above the meninges** (i.e., resting on the dura mater). We have added this clarification in the revised protocol (Section 26, "Electrode Placement and Fixation"). The **meninges were not removed** during the process, and no conductive material was used to enhance signal conductivity, as we determined the natural contact between the electrode and dura mater was sufficient for our recordings. Speak about the impedance of the electrodes.

**Affixing the electrodes to the brain**: The protocol now includes a detailed description of how electrodes were fixed in place. The force applied between the skull and brain was used to maintain stable positioning of the electrodes (Section 27, "Electrode Placement and Fixation"). This is illustrated in the updated diagram, which shows how the electrode was positioned and maintained under the skull until we fixed it permanently. Eventually, it can be firmly fixed by cementing the body of the electrode to the skull.

**Reference Electrode**: We have elaborated on the placement of the reference electrodes on the **cerebellum**. These electrodes were placed at separate cerebellar locations and connected to the noise cancellation system. We also specified how these electrodes were connected to the OpenBCI board for optimal signal recording (Section 6, "Electrode Montage"). **All signal electrodes were referenced to a ground electrode placed on the cerebellum, and no differential signals were compared between pairs of recording electrodes** (Section 6, "Electrode Montage").

**Signal differentiation**: We clarified that the **signal was differentiate**d relative to the ground electrode on the cerebellum. The signal electrodes recorded activity from the brain regions of interest (e.g., frontal lobe, auditory cortex), and these signals were compared relative to the cerebellar ground electrode, not across pairs of recording electrodes.

*d) With respect to electrode placement, please clarify how the tips of the electrodes were positioned relative to relevant landmarks such as the bifurcation of the mid-sagittal sinus. Were all electrodes referenced to the cerebellum, or were there pairs of electrodes across which differential signals were Compared?*

**Response:**

As described in the revised manuscript and on protocols.io, the first electrode was positioned over the HVC (0.2 mm AP, 2.3 mm ML relative to the bifurcation), while the second electrode was positioned over the CMM (2 mm AP, 0.4 mm ML relative to the bifurcation). **All signal electrodes were referenced to a ground electrode placed on the cerebellum, and no differential signals were compared between pairs of recording electrodes** (Section 6, "Electrode Montage").

*e) with respect to electrode placement, can the authors provide any guidance on how much it matters? For EEG, usually, there is quite a bit of dependency of signals on electrode placement. The authors show data from “anterior” and “posterior” electrodes, but do not specify the coordinates where these were placed. We can see that there are some differences between the anterior and the poster electrodes in terms of the signals that are recorded. However, it would be helpful if the authors could provide some more sense of the spatial variation in signals that would help readers understand what kind of spatial resolution is there for distinguishing signals between different brain areas: The authors describe one of the advantages of iEEG as potentially providing a greater “survey“ of activity distribution across the brain than is normally achieved with electrophysiology. This would be substantiated and clarified to the extent that signals from nearby recording locations look different from each other.*

**Response:**

In the new experiments, we placed the electrodes precisely on two important superficial nuclei, HVC and CMM. The exact coordinates of these electrodes are detailed in the protocol and the revised manuscript (Section 24, "Localizing the electrode sites"). Regarding signal localization in our new design, we found some differences between the signals of different electrodes, which have been explained in the result section under the Auditory Response Recording part.*” Interestingly, the ERP response for the BOS differs significantly from the other stimuli; it exhibits a later rise with a smaller slope. This may be due to the distinct responses of these nuclei to the BOS. These differences in response were also distinctly observed in the HVC and CMM. A larger amplitude in the CMM compared to the HVC may result from the dominant role of the CMM in processing auditory stimuli, as is evident across all conditions (Figure 3A, B).”*

We have also mentioned that modifying electrodes can strengthen localization power (see discussion in the article). *Although we observed notable differences between the responses to BOS and reverse BOS in the HVC and CMM electrodes, we should consider that localization can be improved by using higher-impedance electrodes and limiting the contact area. These modifications could also allow us to implant electrodes directly within the nuclei, enabling us to capture LFP signals with greater spatial resolution.*

*f) It would be useful to sketch out more clearly any additional workflow that the authors can provide, for example with respect to how the signals were recorded, and what software was used to process them, etc., so that readers could take advantage of the protocol without needing to reinvent various components or go to other sources. If these components are all provided within Open BCI, that should be clarified with appropriate references.*

**Response:**

**Signal Recording**: The OpenBCI Cyton board was used to acquire signals, and recordings were managed through the OpenBCI GUI. Sections 5, 7, and 13 provide details on setup, electrode connections, and troubleshooting.

**Software and Analysis**: Data was processed in Python using the MNE library for filtering, epoch creation, and ERP analysis. Section 15 provides step-by-step processing details and references.

**References and Guides**: We clarified the use of OpenBCI components and provided direct links to guides and troubleshooting resources. For complete documentation, see Sections 3, 5, and 13.

*2) For the specifics of experimental results that are shown in figures, please also consider where more detail might be helpful for a reader. This again includes some aspects of methods that should be in this paper and not included by reference. For example, for suppression duration shown in figure 2, how was this measured? For low medium, and high levels of isofluorane, how long were animals at a fixed level of anesthesia before measurements were made? For figure 3, is this the average of some number of stimulus presentations or a single trial? What was the stimulus duration in these figures? The stimulus duration is particularly important to show, because of the structure of responses that show, for example, some peak at 1.1 seconds - does that relate in any way to when the stimulus stopped or changed in amplitude? Showing oscillograms of the stimuli here would be helpful and could also address one other question, which is how loud were the stimuli?*

**Response:**

Thank you very much for your accurate comments. More information has been added to the paper overall to provide more detail and value for the reader. Below are examples of information added as an answer to the specific points mentioned.

**Suppression Duration**: We clarified how suppression duration was measured by following the method of Westover et al. (2013), with a defined threshold to segment suppressions and bursts. Details on the stabilization period: Animals were kept at each isoflurane level for **2 minutes**. As reaching the steady state of anesthesia occurs immediately, we used the signal immediately after changing the concentration (Protocol, section 9).

**Stimulus Presentations**: we have provided a detailed description of stimuli (duration, number of repetitions, loudness, spectrogram) (protocol section 11). In the article's discussion section, we have also discussed the features of brain responses and their possible relationships to the stimuli.

*3) as noted at the top, the more that you can show that this set up records useful signals that are similar to those from other eeg recordings in birds, the more likely readers will follow your approach. In that respect, please look a little bit more for songbird references that have recorded EEG’s. Low and Margoliash published a paper (“Mammalian-like features of sleep structure in zebra finches “ PNAS 2008) that I tracked down, but I believe there may be others, including from Low. It would be worth citing this paper and comparing your approach with theirs; if you are in a position to do so, recording activity during sleep to see if you can generate signals like those shown in figure 2 (of Low), and spectra like those shown in figure 3 (differences between slow wave sleep and REM sleep.) would be a nice way of validating your EEG signals relative to those in the literature. It is perhaps beyond the scope of what you might easily do, and is not necessary, but it would also be interesting to see if under light anesthesia you could get different responses to playback of the bird’s own song (BOS) versus reversed (BOS); within the song system nuclei, including the superficial structure HVC, there is a greater response to BOS, and if you are able to detect tuning for song stimuli in this manner or others, that would also promote the utility of these measures for functional use.*

**Response:**

To further validate our method and prove our method’s efficiency, we decided to explore the response’s difference to the BOS and other types of stimuli, as suggested in the comments. We found the suggestion very intriguing and followed it through to add more value to the paper. Therefore, we conducted a new experiment on two birds; before surgery, we recorded their songs to construct BOS. In the recording session, we performed neural recordings on lightly anesthetized birds and presented them with five stimuli: BOS, reversed BOS, conspecific song, a 5 kHz tone, and white noise. We placed our electrodes on the CMM and HVC in this new experiment, two auditory-responsive superficial nuclei. As described in the revised manuscript (Results, Last paragraph), we observed that the response to BOS differed in both electrodes from other stimuli. The response was delayed and predominantly in the Delta band. Additionally, both electrodes showed neural responses to all five stimuli. These are described in the manuscript in more detail, and we believe that by following the reviewer’s suggestions, our data validate the correctness of our protocol.

*Technical: in the versions that I downloaded for review, and the linked figure files, the resolution was very coarse and pixelated. I could not read the axis labels in figure 2, and many other details of the figures were blurry. Please check to make sure that the resolutions of final figures is improved. Note also that Figure 1 was difficult to follow. It looks like there were four colored wires, sticking out in a pitchfork shape from something, but I couldn’t tell what that thing was, and this is not described in the legend. The electrodes are shown as somehow spread out outside of the bird’s brain, but sticking up quite a bit. I have outlined below some more specific suggestions for organizing the protocols.io description. Some of these are redundant with comments above – I was unable to access the protocols.io material from the supplementary materials link, and so wrote the initial review without access to that material. When I was able to access the prorocols.io description, there was something off with the formatting – some sentences were cut off, some strange symbols appeared. I thought the main text was generally well written, but found much of the wording in the prorocils.io to be confusing and to have many typos and grammatical errors, including lots of missing punctuation, and it would benefit from careful proofreading.*

**Response:**

We have modified Figure 3 and provided a comprehensive caption to resolve all of the ambiguity of our illustration. You can find the caption in the article; *“****Figure 1: Graphical abstract of two experiments.*** *Implanted parts on the bird's skull have been identified in different colors. The cream area on the skull shows the cemented region on top of the head. As demonstrated, all other compartments are fixed by cement on the skull. The curly wires represent recording and ground electrodes, and the perpendicularly attached bar (gray) indicates the head post. The electrodes are connected to the Cyton recording board (for a detailed explanation of connections, see the protocol.io). (A) In the first experiment, varying concentrations of isoflurane were used to achieve different depths of anesthesia. (B) In the second experiment, the finch was head-fixed under light anesthesia, and intracranial EEG (iEEG) data were recorded from implanted electrodes during an auditory task.”*. This cartoon illustration is meant to give an overall view to the reader; however, the implanted parts are illustrated as they are, connected to the cemented base and freely accessible at the external end. We used hook clips to connect the external end of the electrodes to the recording board (protocol.io, version 2, step 20, Connecting the electrodes).

**PROTOCOL SECTION**

*For the specific protocol, I suggest that you organize this in sections that separate several different components:*

*0. Overview of OpenBCI and what references readers should access if they are going to follow your protocol. What is provided by OpenBCI and what will readers additionally need to do to implement what you have done? A quick look at the open BCI website indicates that it provides a great deal of useful information. As part of the Over view of the protocol, it would be useful to sketch out what aspects of your recording apparatus and procedures directly use hardware, software, and resources that are well-described by OpenBCI, and what parts you have customized for application to the songbird. Where there are details of the protocol that follow specific step by step instructions form OpenBCI, you do not need to reproduce them, but within your own protocol, you should make clear that the reader should use a specific set of instruction or resources form OpenBCI whenever that is the case. For example, my comments suggestions below may include some things that are redundant with OpenBCI (Such as the pinout configuration, or recording software, to GUI, etc), but there is no clear reference to this in the protocol.io. Overall, it would be fine, if appropriate, to include a statement along the lines “For iEEG recording in the bird, we start with a basic EEG recording configuration that is detailed by OpenBCI. A materials list and step by step directions for this are provided on the OpenBCI website (URL:....). (Then explain what specific OpenBCI documents I need to access and what the goal of this aspect of the protocol is: (will I have a working system that can record EEG signals?, play stimuli? Carry out abalyses? Etc). Then something like: “We suggest readers that are interested in following our protocol first familiarize themselves with these materials from Open BCI before proceeding with customization of the system for songbird experiments as elaborated below” I suggest providing a more complete description of the protocol and ordering it with clearly defined sections along the following lines (doesn’t have to be exactly this, and some of the following may be achieved by specific reference to Open BCI materials, as noted above). It would probably be useful to include a full parts list as a table in this section. IF some of these parts come as. Kit form Open BCI, versus needing to be purchased separately, that could be noted here.*

**Response:**

The following notes were mentioned, as described in comment 1-a. Most of the points are reflected in steps 2, 3, and 4 of the protocol:

In the Overview of OpenBCI section, we have added a description of the OpenBCI system. The OpenBCI Cyton Biosensing Board was used for this study, and we have provided relevant links for obtaining the hardware and software directly from the OpenBCI website. We have also described alternative hardware configurations, such as the Ganglion board. (Section 1, "Getting Started with OpenBCI").

In the Step by step instruction for OpenBCI, we have included step-by-step instructions in the protocol.io description on how to assemble and configure the OpenBCI system for recording iEEG data to make it easier for readers to reproduce our setup. (Section 2, Assembling the Recording Apparatus). In the material section, we also provide a list of all the parts needed to set up the experimental setting and the link or website for purchasing them and their approximate price in a table. (Material section)

*1. Assembling the recording apparatus (this section could explain what kind of inputs and outputs the open BCI system provides for, and give some more specific guidance on how to assemble the relevant recording apparatus: this might include a schematic showing the pins on the open BCI device, and what their functions are (i.e. where should a reference ground be connected? how many EEG electrodes can be connected? Into which pins? does this device itself provide stimulus output or is that something separate? if this device provides for output on what line does that output appear and how would you connect output to speaker or other? If an Opto-isolator is needed, how is that built, or where can it be bought? Schematically, where should it be connected? (Is any of this described on Open BCI site?). What kind of power is required, and where should the power supply be connected to pins on the Open BCI board? (again, if some of this is explained as part of the Open BCI materails, it is fine to refer to that directly, but may still be useful to show us schematic diagram and/or photographs of how you have specifically configured things for the songbird).*

**Response:**

We specifically discuss the electrode montage for recording channels, reference, and noise-canceling in each of the two experiments in steps 4, which also ties into step 2 concerning the board pins. Then, in step 6, we explain how to assemble them onto the board, including some troubleshooting notes about potential interferences. Additionally, essential notes regarding the **battery,** optocoupler, trigger circuit, and checking the Open-BCI trigger input in the software are mentioned in steps 12 and 13.

*2. Electrode assembly; useful to describe as you have done, but perhaps also to show pictures for the recording electrodes, and to make clear to which pins they should be connected. You could also discuss in this section alternative configurations, for example if readers wanted to record more or fewer sites. Is each electron ultimately referenced to the cerebellum? In that case, is the number of recording electrodes anywhere between one and four, or are more EEG electrodes possible? Are the cerebellar electrodes constructed in the same way as the recording electrodes? And are the two pictured cerebellar electrodes the same, just one on each side of the brain, or do they serve some different function? Are those two electrodes linked together if they provide a common ground or do they attach to the open BCI board at separate pins?*

**Response:**

Thank you for your comment. We have provided relevant information in various protocol sections. Specifically, we included an image in Section 4 to illustrate electrode preparation, while details about pin configurations are described in Section 2.4. Additionally, Section 7 offers comprehensive information on maximizing potential recording sites and connecting to reference and noise-canceling pins in the experiment.

*3. Placement of electrodes – this section could both note the specific locations where you placed electrodes for the experiments illustrated in the paper. But it could also discuss issues of placement, such as whether there are particular advantages to placing the electrodes at symmetric locations across the mid-sagittal plane, or any other information you may have gained in experimenting with different electrode locations. What works well and what doesn’t? The Low and Margoliash paper that is mentioned in the main body of the review may also have some useful information on this.*

**Response**

Our new design used different locations for recording electrodes on auditory areas (HVC and CMM). We reported and discussed the differences in acquired signals from these two areas (see the results section in the article, Auditory Response Recording part). As articulated in the protocol section, We implanted our electrodes on one side of the brain. Although we recorded from one side of the brain, recording from both hemispheres can be beneficial for understanding the functional lateralization in bird brains. However, this study aimed to find a method to capture and validate iEEG data from the brains of the zebra finches, and our method satisfies this goal.

Regarding the electrodes' location and signals' differences between different areas, we have reported differences in response to the bird's song compared to the other stimuli (results, Auditory Response Recording): *“Differences are also evident in the HVC and CMM. Specifically, the CMM shows a greater response to conspecific and Reverse BOS stimuli than BOS alone., However, the HVC response to the BOS is more prominent than the reverse BOS. Interestingly, the response to the BOS in HVC is also greater than this response in the CMM. Overall, both areas respond more strongly to song-like stimuli than to white noise or the 5 kHz tone, as seen in the spectrogram. For better illustration, the spectrograms of the presented audio stimuli are shown in Figures 4C and 3C”*

*4. Recording iEEG signals and troubleshooting; Does open BCI come with its own data acquisition software or is this something that is available on the GitHub repository that you list? the GitHub repository is currently empty via the link that I was provided, so I could not evaluate what is there. If there is an open BCI GUI or software for data acquisition, it would be useful to explain how this is set up for your experiments, perhaps also showing a screenshot of the GUI, and providing information about any relevant parameters that need to be set, and where and how files are recorded and can subsequently be opened for analysis. See also the last section about animal handling and procedures – as noted there, it will be important to provide further caveats about the necessity of proceeding only in ways that have been approved by each institutions*

*animal use committee.*

**Response:**

Thank you for bringing this to our attention. The OpenBCI GUI includes built-in recording tools. All necessary information is available in Section 1 of the protocol on Protocol.io. The correct GitHub repository for the project is located at<https://github.com/finch-zebra/ieeg>. The repository now contains the task design, data, and analysis code, as well as the audio stimuli used in the experiments. The provided code includes scripts for preprocessing the iEEG data, such as filtering, event detection, and epoching, suppression duration, ERP, spectrogram and statistical analysis.

*5. Stimulus presentation and data synchronization. Is stimulus presentation part of open BCI, or is this something that you did completely separately with an Arduino, etc.? Here it would also be useful to explain both how you presented stimuli, and how you synchronized presentation with data acquisition. For example, did your Arduino output a TTL pulse or other mark that was recorded on one of the channels of the Open BCI board? Or does the open BCI system drive the Arduino or otherwise control stimulus presentation? You could also include in this section something about how stimuli were generated – what kind of file formats – and how software is set up for this presentation. I don’t think you need to go into this too much, as the requirements for different people using this apparatus will differ, but it is still useful to sketch out some of these details as they will provide a framework for people to modify in order to carry their own experiments.*

**Response:**

The stimulus presentation and synchronization were handled separately from the OpenBCI system, using a Raspberry Pi. The Raspberry Pi controlled the playback of audio stimuli and generated TTL pulses via its GPIO pins to mark the onset and offset of each stimulus. These TTL pulses were recorded on a channel of the OpenBCI board to synchronize the stimulus presentation with iEEG data acquisition.

The OpenBCI system did not drive or control the Raspberry Pi; rather, the Pi operated independently to manage both stimulus timing and synchronization signals. For more details on the task design and synchronization methodology, please refer to section 12 of [protocols.io](https://dx.doi.org/10.17504/protocols.io.q26g7y2w8gwz/v3).

*6. Data analysis. At minimum a description of the file formats and how they can be opened would be useful. Does open BCI itself provide tools for doing things like averaging across presentations and carrying out spectral analysis, or is this something that needs to be done separately? In that case, does your repository include any tools for this, or is this something that people would need to program for themselves in Python or Matlab, etc.? For potential users, providing these details will be very useful and enabling.*

**Response:**

As described in section 15 of [protocols.io](https://dx.doi.org/10.17504/protocols.io.q26g7y2w8gwz/v3), the recorded iEEG data was stored in text format, which can be easily opened and processed using various software tools, such as Python (with libraries like pandas and MNE) or MATLAB.

The OpenBCI software stack wasn’t used for data analysis. We implemented the analysis, such as averaging across stimuli presentations for ERP or performing time-frequency analysis in Python. Further analyses need to be performed separately by the user of our code. Our approach provides a flexible framework that can be easily adapted and extended by users to suit their specific experimental requirements. For details on data processing, please refer to section 15 of [protocols.io](https://dx.doi.org/10.17504/protocols.io.q26g7y2w8gwz/v3) and our repository <https://github.com/finch-zebra/ieeg>.

*7.Troubleshooting. no matter how good your directions, It is likely that things will go wrong for people in one way or another. If you have any advice based on your own experience about troubleshooting problems that have arisen, it could be useful to include some of that here. For example, you experienced some sort of noise issues that necessitated using an opto-isolator, and also a notch filter. Might these be things that are always needed, or perhaps only in cases where there is an unconditioned power supply, etc. Any tips you can provide here will be appreciated when people run into problems. In any of the above sections, it would be fine to direct people to relevant resources provided by open BCI. If there is an open BC manual that covers specific things, you could refer to that more explicitly so that people will know where to look, but I think it would also be good to summarize the points as enumerated above in your protocol itself.*

**Response:**

Regarding troubleshooting, we have added a comprehensive troubleshooting section to the protocol.io file based on our experience. This section includes common issues, such as noise in the signal. Additionally, we have provided guidance on other potential issues, such as **electrode impedance**, **signal saturation**, and **connection problems**. Links to the relevant **OpenBCI troubleshooting resources** have been included for further reference. This section ensures that users have practical solutions for common problems and know where to find additional help if needed (Section 31, "Troubleshooting"). Regarding the optoisolator, we have provided a detailed section in our protocol.io guide in section 14 to assist the reader.

*8. Bird handling and surgical procedures, etc. This is an especially tricky one, but I think should largely be separated from the other sections and treated with care. The current protocol provides lots of details about things like food deprivation, bird handling, inducing anesthesia, placing the bird in a restraining tube, removing feathers, opening craniotomy, delivering analgesics and antibiotics post experiment, housing of birds after experiments, etc. At most institutions, all of these details will need to be approved by the institutional animal care and use committee, and the requirements will be different at different institutions. Many of the specifics outlined here would not be allowed at my institution. For example, I cannot use alcohol to sterilize electrodes, nor can I pluck feathers, but must instead cut them close to the scalp. We would need special permission in order to restrain birds in a tube as you have illustrated, and it might not be allowed. We normally have to provide heat during experiments and recovery. We might not be allowed to use the same antibiotics and analgesics that you list here, etc. For all of these reasons, I think it would probably be easiest for you to write something more general about the animal handling and surgical procedures. You should certainly note that any specifics need to be approved by each users institutional animal use committee. If you do that, I suppose you could outline the specifics of what you are allowed to do at your institution, but please make it clear that this might not be universally accepted. I am not sure for example that we would be allowed to use ear bars of the sort that you describe -- made from hypodermic needles, and the mask that we use for isoflurane anesthesia would need to be specifically approved by our veterinary staff, etc. The one thing that I think is worth making specific is exactly how your electrodes are placed - in this case, inserting them into the craniotomy, but then sliding them between dura and the skull. It would helpful to be similarly specific about how the cerebellar electrodes are placed.*

**Response:**

In response to this comment, we revised the protocol to provide more general guidance on these procedures. We indicated that each institution's relevant **Institutional** **Animal** **Care** **and** **Use** **Committee** **(IACUC)** must approve all specific practices. To help users identify the sections that may require special approval, we have marked them with the **¥ symbol**. These include practices such as **food deprivation, bird handling, anesthesia, post-experimental care, and ear bars** (Sections 21–31). This notation ensures that users know the need for institutional approval and that these procedures may not be universally accepted.

Additionally, we have clarified the details of electrode placement and positioning between the **dura and skull** and the placement of **cerebellar reference electrodes** (Section 27, "Electrode Placement and Fixation").

Regarding the ear bar adopters, we have provided a detailed explanation of their preparation (Section 19: “Constructing ear bar adaptor”), which has been reviewed by our veterinary staff before. The ear bar adopter construction method ensures the investigator that the fixating method is not traumatic.

Additionally, the ethics statement has been reported in the Guidelines and protocol part of the protocol.io: “ *All procedures were approved by the Research Ethics Committee of the School of Cognitive Sciences (SCS) of the Institute for Research in Fundamental Sciences (IPM, protocol number 1402/40/1/2841*)”.

As mentioned in the above paragraphs, the exact location of the electrodes regarding the bifurcation and cortex has been declared in the protocol section (section 26: Electrode placement and fixation).

# Response to Reviewer 2’s comments:

*• The increase in duration of suppression with higher doses of anesthesia is robust. A few concerns and suggestions are listed here*

*◦ The authors should briefly explain how they calculated the duration of suppression. Currently they cite an existing paper and say that they followed this method. They should still briefly describe the calculation while including the citation. This allows the reader to get a sense of the method without having to go to the previous citation. The reader can go to the citation if they want more details.*

**Response:**

In respond to raised concern, in the revised manuscript, we address this comment by briefly describing an alternative method for computing suppression duration and elaborating on the method used in the cited paper in greater detail in the Methods section of revised manuscript, as outlined below.

“*In 2013, Westover et al. introduced a new method involving an empirical approach, in which two experienced electroencephalographers manually segmented EEG data based on visual pattern recognition and clinical judgment. Additionally, the study developed an automatic segmentation method using recursive variance estimation, which classifies bursts and suppressions based on the adaptive mean and variance of the EEG signal, optimized to match expert consensus. As a result, a real-time algorithm was created to automatically differentiate suppressions from non-suppressions (bursts) in EEGs. Using this algorithm, we rely on the total suppression duration for each task condition and compare these durations statistically. At each level of isoflurane (low, medium, and high), we compute the suppression duration for each 2-minute segment of the session and perform statistical analyses to compare the conditions (16).”*

*◦ Currently statistics are done in a pairwise fashion. This involves multiple comparisons and the p-value should be adjusted for multiple comparisons (*[*https://en.wikipedia.org/wiki/Multiple_comparisons_problem#Controlling_procedures*](https://en.wikipedia.org/wiki/Multiple_comparisons_problem#Controlling_procedures)*). It would be best to adjust p-values using the Bonferroni correction.*

**Response:**

Thank you for raising this important note, in the revised version as our data wasn’t normally distributed, we ran Friedman non-parametric test following by pairwise comparison with Bonferoni correction. These changes made in the analysis and result sections of the revised manuscript. The following is added to explain the new statistics analysis.

*“To compare suppression durations across the three experimental conditions, a Friedman test was conducted as a nonparametric alternative for repeated measures. When significant differences were found, post hoc pairwise comparisons were performed using Wilcoxon signed-rank tests with Bonferroni correction to adjust for multiple comparisons.”*

*◦ The authors should also confirm that the bird's responses were also related to the level of anesthesia since there can be some inter-individual variability in response to anesthesia. Therefore, classifying the level based on concentration of isofluorane alone may not be enough.*

**Response:**

Thank you for this important suggestion. To address this, we adjusted a wide range of isoflurane levels and categorized them into three groups (low, medium, and high) based on both concentration and observed physiological responses during recording. This approach helps minimize inter-individual variability and ensures that the bird's responses are indeed related to the level of anesthesia. Additionally, the minimal standard error within each group and the large p-values in the comparison of variability between groups further confirm the robustness of this categorization and its ability to account for inter-individual differences effectively. However, to be clearer about the raised concern we compute and add the minimum and maximum of duration besides the average and se to ensure that the inter-individual variability is not considerable. Here is the summary of this results.

| **Region** | **Condition** | **Mean** | **Min** | **Max** | **SE** |
| --- | --- | --- | --- | --- | --- |
| Anterior | Condition 1 (Low) | 8.83 | 0.00 | 58.70 | 5.96 |
| Anterior | Condition 2 (Medium) | 42.32 | 16.29 | 80.00 | 8.45 |
| Anterior | Condition 3 (High) | 54.87 | 37.28 | 77.14 | 4.03 |
| Posterior | Condition 1 (Low) | 4.03 | 0.00 | 12.05 | 1.47 |
| Posterior | Condition 2 (Medium) | 48.30 | 21.06 | 80.00 | 8.24 |
| Posterior | Condition 3 (High) | 59.18 | 43.34 | 76.17 | 4.04 |

*• There is no quantification of differences in EEG responses to auditory stimuli. This is only qualitatively described. It is ok if this is just to show that the method does work. However, it would be useful to know where the electrodes were exactly. Given that this is iEEG responses, which I am assuming is intra-cranial EEG from the surface of the brain it would be useful to know where these electrodes are. This would help understand what kinds of responses to expect.*

**Response:**

In the revised version we mention the a exact locations of electrode sites in the experiment two were the location is more important as we have auditory stimuli, further in analysis we add new figure to compare spectrogram of task condition to provide better explanation for the experiment.

Regarding your point about iEEG is stand for intracranial EEG.

• *The authors need to compare their recordings with other methods for recording EEG (or recordings from other papers) to validate their method. In other words, are they really recording EEG signals and are their recordings similar to what has been described in the literature.*

**Response:**

Thank you for raising this important comment. To address this core aspect of the method, we conducted a new experiment to obtain comparable results with those seen in the literature. In this experiment, we precisely placed the electrodes on two key superficial nuclei, HVC and CMM. We recorded two bird songs to construct BOS and performed neural recordings on lightly anesthetized birds while presenting them with five stimuli: BOS, reversed BOS, conspecific song, a 5 kHz tone, and white noise. As described in the revised manuscript (Results, last paragraph), we observed that the response to BOS differed from other stimuli in both electrodes, with delayed responses predominantly in the Delta band. Additionally, both electrodes showed neural responses to all five stimuli. These results are described in more detail in the manuscript, and we believe that by following the reviewer’s suggestions, our data validate the correctness of our protocol.

*• An important aspect of the discussion is for the authors to explain how this technique could be useful and this is not explored. This could also be useful for characterizing sleep states.*

**Response:**

Thank you for your comment. We address this in the discussion part, along with other possible capabilities of this method; we have also focused on the sleep studies on zebra finch and explained how our method could be helpful in these studies.

“One of the early and valuable uses of EEG signals is detecting sleep states and disorders (28,29). Low et al. have studied sleep in zebra finches and found mammalian-like patterns of brain activity in them during sleep (30). The effect of sleep on developing song memory has been studied previously, and this phenomenon reveals an evolutionary fundamental rule of sleep in memory consolidation (31,32). Our method can be utilized for studying sleep in zebra finches. The chronic recording ability of this method makes it suitable for studying the rule of sleep on song memory during development and the long-term effect of sleep deprivation in adults”

*• The authors do not define what "iEEG" is - I assumed it is intracranial EEG, but the authors should define this the first time they use the term. Is it not possible to record this from the skull?*

**Response:**

Thank you for your comment. In the revised manuscript, we have addressed this by clearly defining "iEEG" at the beginning of the introduction and in the title. Recording from the skull is not as effective because the intervening layers attenuate the signal and distort the recorded activity. However, it is also possible to apply EEG, but the results are not as fine or precise as those obtained through iEEG.

*• Why is this method superior to regular EEG recordings from the skull - possibly from on top of the second layer of skull? Again the introduction should really provide more information on the limitations of current methods and why this method is likely to be useful.*

**Response:**

iEEG provides superior spatial and signal resolution compared to scalp EEG by directly recording from the brain, avoiding distortion from the skull. Furthermore, iEEG's proximity to LFP allows for more accurate, reliable data that can be generalized to brain activity, boosting confidence for more sophisticated recordings. Additionally, iEEG enables precise localization of brain regions, making it highly suitable for tasks that require detailed spatial information during recording sessions.

*• Another useful addition would be to provide a table with costs for the entire system and compare this with costs for commercially available systems - this would support the claim that this is an affordable solution.*

**Response:**

Added in the material section of protocol.io.

*• Typo - "ground" electrodes are referred to as "grand" electrodes in the protocol.*

**Response:**

Corrected in the new version of protocol.io
